# Adaptation and validity of the Sleep Quality Scale among Chinese drivers

**DOI:** 10.1371/journal.pone.0259813

**Published:** 2021-11-11

**Authors:** Shuang Chen, Long Sun, Changlu Zhang

**Affiliations:** 1 School of Psychology, Liaoning Normal University, Dalian, Liaoning, P. R. China; 2 School of Education, Shenyang Normal University, Shenyang, Liaoning, P. R. China; Tongii University, CHINA

## Abstract

**Purpose:**

Poor sleep quality is closed related with driving accidents. However, due to the lack of a valid instrument for assessing drivers’ sleep quality, few studies have examined drivers’ sleep quality and its associations with driving behaviours and traffic accidents in China. The aim of this paper is to revise the Sleep Quality Scale (SQS) and assess its reliability and validity in Chinese drivers.

**Methods:**

522 Chinese drivers aged from 18 to 56 years old agreed to complete the SQS, Daytime Sleepiness Perception Scale version 4 (DSPS-4), Self-report of Risky Driving Behavior (RD-SR) and Self-assessment of the Likelihood of Being Involved in a Risky Driving Situation (RD-SA).

**Results:**

The final Chinese version of the SQS contained 23 items across four factors: difficulty in getting up, difficulty in falling asleep, sleep recovery and daytime dysfunction. Second, man scored lower on the difficulty in falling asleep factor but higher on the sleep recovery factor than women. Third, low to moderate correlations were found between the SQS factors and the DSPS-4, RD-SA and RD-SR, indicating that the validity of the revised scale was satisfactory. More importantly, daytime dysfunction factor is an effective predictor of violation involvement and accident involvement.

**Conclusion:**

The revised SQS has acceptable reliability and validity and can be used as a tool to measure the sleep quality of Chinese drivers.

## Introduction

Sleep problems have an important influence on individuals’ physical and mental health around the world. Sleep-related problems among drivers result in many risky driving behaviours and traffic accidents [1–4]. According to the Transportation Safety Authority of China, sleepiness while driving or sleep loss is a major cause of traffic accident-related death. In 2016, 787 traffic accidents occurred in the Huai’an section of the Beijing Shanghai Expressway, including 414 traffic accidents caused by sleepy driving, accounting for 52.6% of the total number of accidents [5]. However, due to the lack of a valid instrument for assessing Chinese drivers’ sleep quality; few studies have examined drivers’ sleep quality and its associations with driving behaviours and traffic accidents. Therefore, the present study aimed to develop a valid instrument for assessing Chinese drivers’ sleep quality and to examine its reliability and validity.

### Sleep quality and driving behaviours, traffic accidents

Good sleep quality is crucial for driving safety, as it can provide drivers with adequate energy to avoid risky driving behaviour. Many studies have found that poor sleep quality or sleep loss while driving can lead to more distracted driving [6], greater variation in steering, speed and lane position [7, 8], and slower detection of road hazards [9]. Only one study has found a positive correlation between sleep loss and traffic accidents among Chinese drivers [10]. However, to the best of our knowledge, few studies have examined the relationships between drivers’ sleep quality and the frequency of risky driving behaviour and drivers’ involvements in risky driving situations.

Poor sleep quality among drivers is also closely related to traffic accidents [1, 2, 4]. For example, one study found that half of Canadian drivers reported feeling sleepy while driving, 31% reported falling asleep while driving, and 12% reported having had a traffic accident due to sleepiness [11]. A survey conducted in 19 European countries also found that among drivers who fell asleep, the median prevalence of sleep-related accidents was 7.0% [2]. However, previous studies did not examine the characteristics of sleep quality of drivers with and without traffic violations or traffic accidents.

### Sleep Quality Scale

To explore sleep quality among individuals and its possible influencing factors, many instruments have been developed, such as the multiple sleep latency test [12], Pittsburgh Sleep Quality Index [13], Stanford Sleepiness Scale [14] and Sleep Quality Scale. Prior studies have shown that although objective tools, such as the MSLT, are effective in diagnosing individuals’ sleep quality, they are not as convenient and inexpensive as self-reported scales [15]. More importantly, multidimensional sleep scales, such as the SQS, can assess sleep quality from multiple perspectives to comprehensively reflect the relationship between sleep and other psychological variables, such as well-being and traffic accidents [16]. Given the relationships between sleep quality and driving behaviours and accidents [1, 3], this study aims to adapt the SQS to Chinese drivers.

The SQS was developed in South Korea in 2006. It is one of the most popular self-evaluation sleep quality scales, and it mainly assesses the sleep status of the respondents in the past month. The 28-item scale mainly evaluates the sleep quality of individuals across six factors: daytime dysfunction (representing symptoms resulting from poor sleep), restoration after sleep (the primary function of sleep), difficulty in falling asleep (sleep initiation), difficulty in getting up, satisfaction with sleep (the level of overall gratification and sufficient sleeping time) and difficulty in maintaining sleep (awakening during sleep). The SQS showed satisfactory reliability in a Korean sample with obstructive sleep apnoea syndrome (OSAS) [17].

### Aims of the present study

The main purpose of this study is to adapt the SQS for Chinese culture and the driving environment. Exploratory factor analysis (EFA) and confirmatory factor analysis (CFA) were used to assess the reliability of the Chinese version of the SQS (SQS-C), and the associations between the SQS-C factors, the DSPS-4 and risky driving behaviours were used to evaluate the validity of the scale. The second aim is to determine whether the SQS-C factors can predict risky driving behaviour or distinguish drivers with and without traffic accidents. It was predicted that difficulty in getting up, difficulty in falling asleep, sleep recovery and daytime dysfunction are positively correlated with the score of Daytime Sleepiness Perception Scale-4 (DSPS-4). All four factors of the SQS-C are positively correlated with the score of Self-report of Risky Driving Behaviours (RD-SR) and Self-assessment of the Likelihood of Being Involved in a Risky Driving Situation (RD-SA).

## Methods

### Participants

The present study was approved by the Logistics Department for Civilian Ethics Committee of Liaoning Normal University. Five hundred and twenty-two private car drivers were randomly recruited from Dalian, Shanghai and Chengdu. Drivers were randomly divided into two equal-sized samples, each had 261 drivers. However, data from seven drivers were discarded due to missing answers. The final analysis contained data from 515 drivers.

Sample one included 256 drivers, including 120 men (46.88%) and 136 women (53.12%). The participants ranged in age from 18 to 56 years. The participants’ years of driving experience ranged from 3 months to 30 years. The participants’ average driving hours per week ranged from 0.25 to 8.50 hours. Regarding traffic violation history, 52 participants reported that they had traffic violations in the last 12 months, and 50 participants had traffic accidents in the last 12 months.

Sample two included 259 drivers, including 119 men (45.9%) and 140 women (54.1%). The participants ranged in age from 18 to 56 years. The participants’ years of driving experience ranged from 3 months to 30 years. The participants’ average driving hours per week ranged from 0.25 to 8.50 hours. Regarding traffic violation history, 50 participants reported that they had traffic violations in the last 12 months, and 49 participants had traffic accidents in the last 12 months. Detailed demographic information is shown in Table 1.

**Table 1:** Demographic information.

Demographic variables	Sample 1(*n* = 256)				Sample 2(*n* = 259)				*p*
	Min	Max	Mean	SD	Min	Max	Mean	SD	
Gender (Women/Men)	136/120				140/119				0.45
Age (yr)	18	56	27.84	9.39	18	56	34.66	11.97	0.01
Years of driving experience (yr)	0.25	30	3.55	5.06	0.25	30	5.89	7.31	0.05
Average driving hours per week	0.25	8.50	2.83	2.10	0.25	8.50	2.68	2.06	0.41
Number of violations last 12 months	0	3	0.27	0.60	0	2	0.26	0.57	0.83
Number of accidents last 12 months	0	3	0.25	0.56	0	2	0.22	0.48	0.57

### Measures

#### Sleep Quality Scale

The original SQS contained 28 items across 6 dimensions: daytime dysfunction (12 items), restoration after sleep (4 items), difficulty in falling asleep (4 items), difficulty in getting up (3 items), satisfaction with sleep (3 items) and difficulty in maintaining sleep (2 items) [18]. Participants were asked to rate each item on a 4-point Likert scale ranging from few (0) to almost always (3). Notably, the items on the “restoration after sleep” and “satisfaction with sleep” factors were reverse scored.

The SQS was translated into Chinese with the permission of the rights holder of the scale. The original items were first translated by a Chinese researcher, and this version was then translated into English by another qualified researcher following the procedure of translation/back translation. After amending the wording of some items, an initial 28-item scale was obtained.

#### Dangerous Driving Behavior Scale

The Dangerous Driving Behavior Scale had 28 items across two subscales: the Self-report of Risky Driving Behavior (RD-SR) and the Self-assessment of the Likelihood of Being Involved in a Risky Driving Situation (RD-SA) [19]. The RD-SR is a 5-point Likert scale ranging from never (1) to very often (5). The RD-SR mainly measures risky driving behaviours. The RD-SA is a 4-point Likert scale ranging from highly unlikely (1) to very likely (4). The RD-SA asks drivers to evaluate the possibility of entering into different risky driving situations (such as speeding and car racing). In this study, the internal reliability of the RD-SR was 0.96, and that of the RD-SA was 0.93. Studies have shown that sleep quality is closed related to driving behaviours and traffic accidents [1, 2, 10], hence the RD-SR and RD-SA were used to validate the SQS in this study.

#### Daytime Sleepiness Perception Scale-4 (DSPS-4)

The DSPS-4 was developed by Marques in 2017 [20]. The 4-item scale was used to evaluate the subjective perception of sleepiness. The DSPS-4 is a 5-point Likert scale ranging from never (0) to always (4). The total score is obtained by averaging the scores of four items. The Cronbach’s alpha for DSPS-4 was 0.57 in this study. Given that daytime sleepiness is one of the results that derived from poor sleep quality [21], DSPS-4 was used to validate the SQS in this study.

#### Demographic questionnaires

Participants were also asked to provide demographic information, including gender, age, years of driving, number of traffic violations in the last 12 months, average driving hours per week, and number of traffic accidents in the last 12 months.

### Procedure

The SQS, DSPS-4, RD-SR and RD-SA were administered to 522 Chinese drivers in supermarket like Warmart and Carrefour in Dalian, Shanghai and Chengdu. Data were collected from November 26 to December 10, 2020. All participants signed informed consent forms and were required to complete all scales within 30 minutes. Upon completion, each participant received a gift coupon (10 RMB).

### Data analysis

The data were analysed using SPSS version 18.0. First, this study examined the psychological properties of the items, including the mean, standard deviation and corrected item-total correlation (see Table 2). Second, we conducted EFA (sample 1) and CFA (sample 2) to explore the factorial structure of the SQS. Third, reliability analysis was conducted to examine the internal consistency of the SQS-C factor with a Cronbach’s alpha value greater than 0.6 expected. Fourth, Pearson’s correlation analyses and independent sample t-tests were conducted to examine the effects of demographic variables on the SQS-C factors using the whole sample. Then, the associations among the SQS-C factors, the RD-SR, the RD-SA and DSPS-4 were analysed to explore the construct validity of the SQS-C. The role of the SQS-C factors in the prediction of the RD-SR and RD-SA were examined using hierarchical regression analysis with enter methods. Demographic variables entered in the first step and then the four SQS-C factors entered separately during the regression analysis. Finally, the predictive role of the SQS-C factors in whether a driver involved in traffic violations or accidents were examined using binary logistic regression analysis.

**Table 2:** Descriptive statistics of SQS-C items (*n* = 256).

Items	Mean	SD	Item test	Factor loading
1. Refreshed feeling of body after sleep	2.21	0.96	0.41**	0.79
2. Enough sleep time	2.49	1.01	0.46**	0.73
3. Regaining vigor after sleep	2.13	1.00	0.51**	0.86
4. Relief of fatigue after sleep	2.16	1.00	0.32**	0.80
5. Satisfaction with sleep	2.25	0.99	0.50**	0.77
6. Clear-headed feeling after sleep	2.20	0.96	0.40**	0.80
7. Difficulty in falling asleep	1.46	0.76	0.57**	0.61
8. Difficulty in getting back to sleep after nocturnal awakening	1.41	0.72	0.48**	0.79
9. Tossing and turning sleeplessly	1.56	0.79	0.60**	0.67
10. Never falling asleep after awakening during sleep	1.43	0.70	0.54**	0.80
11. Feeling unlikely to sleep after sleep	1.58	0.87	0.41**	0.73
12. Wish for more sleep after getting up	2.52	1.10	0.37**	0.83
13. Difficulty in getting up after sleep	1.85	0.93	0.43**	0.69
14. Decrease of appetite due to poor sleep	1.49	0.74	0.58**	0.59
15. Difficulty in thinking due to poor sleep	1.78	0.92	0.64**	0.77
16. Decrease of interest in work or others due to poor sleep	1.90	0.94	0.65**	0.80
17. Increase of mistakes due to poor sleep	1.68	0.84	0.60**	0.83
18. Increase of forgetfulness due to poor sleep	1.92	0.92	0.70**	0.77
19. Difficulty in concentrating due to poor sleep	1.90	0.91	0.67**	0.85
20. Sleepiness that interferes with daily life	1.67	0.81	0.67**	0.64
21. Decrease of desire due to poor sleep	1.51	0.75	0.69**	0.75
22. Getting tired easily at work due to poor sleep	1.94	0.94	0.67**	0.78
23. Painful life due to poor sleep	1.59	0.82	0.64**	0.68

Note

*p < 0.05

**p < 0.01.

## Results

### Exploratory factor analysis

To explore the factorial structure of the SQS, EFA with principal component extraction and varimax rotation was conducted using sample 1. The results revealed four factors that explained 62.34% (KMO = 0.90, Bartlett = 4198.64, *p* < 0.001). Two items generated a new factor with a Cronbach’s alpha coefficient lower than 0.70, and another three items had the same loading on more than one factor and thus were discarded. Of the 5 discarded items, 2 items belonged to the original daytime dysfunction factors, 2 items belonged to the original difficulty in maintaining sleep factor and 1 item belonged to satisfaction with sleep factor. Notably, the items of the original satisfaction with sleep factor merged into sleep recovery factor in this study.

The next 23 items were submitted to a second EFA. The results revealed a four-factor structure that explained 63.34% of the variance (KMO = 0.89, Bartlett = 3179.45, *p* < 0.001). The four factors were difficulty in getting up (2 items), difficulty in falling asleep (5 items), sleep recovery (6 items) and daytime dysfunction (10 items), which explained 31.42%, 17.69%, 8.39% and 5.84% of the variance, respectively. The factor loadings were shown in Table 2.

### Confirmatory factor analysis

CFA was conducted to examine the structural fitness of the revised SQS via Amos version 21.0. A *X^2^/df* value less than 3 and an RMSEA (root mean square error of approximation) value less than 0.10 are acceptable. Additionally, the cut-off value for the CFI and TLI were set at 0.9 [22]. The present study first examined the model fit of the original SQS. However, the values of CFI and TLI were less than 0.90 (*X^2^* = 788.633, *df* = 335, *X^2^/df* = 2.354, CFI = 0.86, TLI = 0.84, RMSEA = 0.08). Next, the model fit of the factorial structure obtained through the EFA was examined. The results showed that the model fit indices were acceptable (*X^2^* = 509.141, *df* = 224, *X^2^/df* = 2.273, CFI = 0.91, TLI = 0.90, RMSEA = 0.07). The path diagram is shown in S1 Appendix.

### Reliability of the SQS-C

Reliability analysis showed that the Cronbach’s alpha coefficient for the whole scale was 0.89, and 0.82 for the difficulty in falling asleep factor, 0.60 for the difficulty in getting up factor, 0.88 for the sleep recovery factor, and 0.93 for the daytime dysfunction factor. The results are similar to the findings of the original scale. In the original SQS [18], the Cronbach’s alpha coefficient for the factors ranged from 0.61 to 0.90.

### Gender differences in the SQS-C factors

Independent sample t-tests showed (see Table 3) that man had lower scores on the difficulty in getting up factor than women, while women had lower scores on the sleep recovery factor than man. No gender differences were found in the scores of the difficulty in falling asleep and daytime dysfunction factors.

**Table 3:** Gender differences in the SQS-C factors (*n* = 515).

SQS-C factors	Men (*n* = 239)		Women (*n* = 276)		*t*	*Cohen’s d*
	M	SD	M	SD		
Difficulty in getting up	2.06	0.83	2.30	0.87	-3.27**	-0.28
Difficulty in falling asleep	1.49	0.57	1.48	0.61	0.23	0.02
Sleep recovery	2.32	0.81	2.16	0.75	2.28*	0.20
Daytime dysfunction	1.72	0.68	1.76	0.66	-0.74	-0.06

Note

*p < 0.05

**p < 0.01.

### SQS-C factors and age, years of driving experience and weekly driving hours

Pearson correlations revealed that age and years of driving experience were negatively correlated with difficulty in getting up (*r* = -0.30, *p* < 0.01; *r* = -0.27, *p* < 0.01), sleep recovery (*r* = -0.19, *p* < 0.01; *r* = -0.16, *p* < 0.01) and daytime dysfunction (*r* = -0.22, *p* < 0.01; *r* = -0.19, *p* < 0.01). The results suggest that the older the drivers become and the more years of experience they have, the less likely they are to experience poor sleep quality. No significant associations between average driving hours per week and the SQS-C factors were found.

### SQS-C factors and DSPS-4, RD-SR and RD-SA

Correlations among the SQS-C factors, the DSPS-4, the RD-SR and the RD-SA are shown in Table 4.

**Table 4:** Correlations between SQS-C factors, DSPS-4 and driving behaviours (*n* = 515).

Variables	Difficulty in getting up	Difficulty in falling asleep	Sleep recovery	Daytime dysfunction
Difficulty in falling asleep	0.20**			
Sleep recovery	0.02	0.23**		
Daytime dysfunction	0.42**	0.46**	0.10*	
RD-SA	0.15**	0.28**	0.13**	0.30**
RD-SR	0.15**	0.33**	0.16**	0.30**
DSPS-4	0.37**	0.21**	-0.02	0.34**

Note

*p < 0.05

**p < 0.01.

DSPS-4: Daytime Sleepiness Perception Scale-4

RD-SA: Self-assessment of the Likelihood of Being Involved in a Risky Driving Situation

RD-SR: Self-report of Risky Driving Behaviour.

All four of the SQS-C factors were significantly correlated with the RD-SA and the RD-SR. Three factors, i.e., daytime dysfunction, difficulty falling asleep, and difficulty in getting up, were significantly correlated with the DSPS-4. The results indicated the construct validity of the SQS-C was acceptable.

### Predictive role of the SQS-C factors on driving behaviours

To explore the predictive role of the four SQS-C factors on RD-SA and RD-SR, two regression analyses with enter methods were performed using the RD-SA and the RD-SR as dependent variables and the four SQS-C factors as independent variables after controlling for demographic factors. The results are shown in Table 5.

**Table 5:** Regression coefficients for the prediction of driving behaviours (*n* = 515).

Variables	RD-SA		RD-SR	
	*β*	*t*	*β*	*t*
Gender	-0.202	-4.792**	-0.150	-3.592**
Age	-0.074	-1.181	-0.142	-2.303*
Years of driving experience	0.044	0.752	0.069	1.183
Average driving hours per week	-0.046	-1.065	-0.044	-1.027
*F*	7.151**		6.690**	
*ΔR^2^*	0.046		0.042	
**Difficulty in getting up**	0.050	1.078	0.038	0.810
*F*	8.354**		7.514**	
*ΔR^2^*	0.021		0.018	
**Difficulty in falling asleep**	0.164	3.480**	0.222	4.759**
*F*	13.460**		15.640**	
*ΔR^2^*	0.060		0.086	
**Sleep recovery**	0.045	1.052	0.058	1.351
*F*	11.665**		13.651**	
*ΔR^2^*	0.000		0.001	
**Daytime dysfunction**	0.200	4.001**	0.167	3.378**
*F*	12.510**		13.616**	
*ΔR^2^*	0.025		0.017	
Total *ΔR^2^*	0.152		0.164	

Note

*p < 0.05

**p < 0.01

RD-SR: Self-report of Risky Driving Behaviour.

RD-SA: Self-assessment of the Likelihood of Being Involved in a Risky Driving Situation.

Table 5 showed that the effects of difficulty in falling asleep and daytime dysfunction on the RD-SA and RD-SR are significant. These factors can explain 10.6% of the variance in RD-SA and 12.2% of the variance in RD-SR.

### SQS-C factors and traffic violations

The number of traffic violations and accidents in the last 12 months were significantly correlated with the difficulty in falling asleep (*r* = 0.09, *p* < 0.05; *r* = 0.12, *p* < 0.01) and daytime dysfunction factors (*r* = 0.12, *p* < 0.05; *r* = 0.13, *p* < 0.01).

Next, the predictive role of the SQS-C factors in whether a driver involved in traffic violations or accidents was examined using binary logistic regression analysis. A total of 102 drivers had traffic violations in the past year, while 413 had no traffic violations. Ninety-nine of the participants reported at least one accident, while 416 did not have traffic accidents. Demographic variables entered as controlled variables and the SQS-C factors entered as independent variables. The results (see Table 6) show that daytime dysfunction is effective predictor of violation involvement (OR = 2.119, 95%CI = 1.386–3.247) and accident involvement (OR = 2.373, 95%CI = 1.529–3.683).

**Table 6:** Predictive role of the SQS-C factors on violation and accident involvement.

Variables	Violation involvement			Accident involvement		
	Odds ratio	95% CI	*p*	Odds ratio	95% CI	*p*
Gender	1.276	0.781–2.085	0.330	1.531	0.929–2.521	0.095
Age	1.05	1.018–1.083	0.002	1.057	1.024–1.091	0.001
Years of experience	1.609	1.021–1.120	0.005	1.054	1.006–1.103	0.026
Driving hours weekly	0.986	0.865–1.124	0.831	1.027	0.901–1.171	0.687
Difficulty in getting up	0.963	0.609–1.345	0.827	0.735	0.517–1.046	0.087
Difficulty in falling asleep	1.285	0.841–1.964	0.247	1.325	0.862–2.035	0.199
Sleep recovery	0.906	0.650–1.262	0.558	1.036	0.745–1.441	0.832
Daytime dysfunction	2.119	1.386–3.241	0.001	2.373	1.529–3.683	0.001

## Discussion

This study was the first to translate and adapt the Sleep Quality Scale to Chinese drivers and examined its reliability and validity. The factorial structure of the SQS was examined through EFA and CFA, and its associations with demographic factors, driving behaviours and traffic violations were investigated. The results indicate that the reliability and validity of the Chinese version of the SQS were satisfactory.

First, the final version of the SQS-C consists of 23 items, which can be divided into 4 factors: difficulty in getting up, difficulty in falling asleep, sleep recovery and daytime dysfunction. The SQS-C has a factorial structure similar to that of the original SQS [18]. However, according to the results of the first exploratory factor analysis, the items of the original 2 factors were merged into one factor. The present study found that the difficulty in getting up, difficulty in falling asleep and daytime dysfunction factors were positively correlated with each other. Contradictory to the findings of a previous study [18], no significant correlation was found between the difficulty in getting up and sleep recovery factors in the present study. This may be due to the small sample size. Except for the difficulty in getting up factor, all the other three factors have Cronbach’s alpha coefficients greater than 0.8, which indicates that the SQS-C has satisfactory reliability.

Second, the present study found that men scored lower on the difficulty in getting up factor but higher on the sleep recovery factor than women, suggesting that men are more likely to get up early and less likely to feel good in sleep recovery than women do. These findings are consistent with the findings of previous studies showing that men are more likely to report good sleep quality than women [23]. Negative correlations were found among age, years of driving experience and the difficulty in getting up, sleep recovery and daytime dysfunction factors. The results suggested that the more years of driving people have or the older the drivers are, the worse their sleep quality will be. In line with the findings of an Iranian study [24], the present study found that the correlations between average driving hours per week and the four factors of the SQS-C were not significant. One possible explanation is that the sample size in the present study is small. Hence, future studies should further explore the possible influences of average driving hours per week on sleep quality.

Furthermore, significant associations among the SQS-C factors, the DSPS-4, the RD-SR and the RD-SA indicated that the discriminant validity of the scale was acceptable. Notably, significant correlations were found between DSPS-4 and SQS-C factors, but the coefficients were relatively small. It is possible that, on the one hand, daytime sleepiness is just one of the indicators of poor sleep quality [21]. On the other hand, drivers may not be able to adequately report sleepiness because they are not fully aware of it and that social desirability may also cause drivers to report less sleepiness [25]. Regression analysis showed that scores on the difficulty in falling asleep and daytime dysfunction factors could predict the frequency of risky driving behaviours and the likelihood of being involved in risky driving situations. The results suggested that drivers with poor sleep quality, such as difficulty in falling asleep and daytime dysfunction, might be more prone to risky driving behaviours. This is similar to the findings of previous studies showing that more risky driving behaviours were found in obstructive sleep apnoea drivers [16, 17].

Finally, significant correlations were found between the number of traffic violations and accidents and the difficulty in falling asleep and daytime dysfunction factors. More importantly, drivers with traffic violations and accidents scored higher on the difficulty in falling asleep and daytime dysfunction factors than drivers who had no violations and accidents. One explanation is that poor sleep quality results in increased blink duration [8, 26], which in turn might jeopardize a driver’s ability to detect and respond to hazards in time [9]. The findings are of great importance in providing evidence for the usefulness of the SQS-C in driver assessment.

## Limitations

There are limitations in the present study. One limitation is that because of the use of self-report methods, the associations between the SQS-C factors and risky driving behaviours might be affected by social desirability. However, the main aim of the present study was to examine the validity and reliability of the revised SQS. As in previous studies, self-reported methods were suitable for achieving these goals [18]. Another limitation is that few of the participants had been involved in risky driving behaviours in the driving of past years. Follow-up research with a larger sample is needed to further explore the correlations between risky driving behaviours and the Chinese version of the SQS.

## Conclusion

The findings of the present study indicated that the Chinese version of the SQS has sufficient psychometric properties and is a valid and reliable tool for assessing Chinese drivers’ sleep quality. The present study is the first to reveal that drivers’ frequency of risky driving behaviours is associated with difficulty in getting up, falling asleep and daytime dysfunction. Moreover, daytime dysfunction can significantly predict drivers’ violation involvement and accident involvement. The findings not only support the usefulness of the SQS in driver population, but also can help develop some interventions for drivers whose sleep quality is poor.

## Supporting information

S1 Appendix Path diagram obtained through confirmatory factor analysis. Note: **digu**: difficulty in getting up; **difa**: difficulty in falling asleep; **sleepy**: sleep recovery; **dd**: daytime dysfunction.
(TIF)

S1 File Chinese version of the SQS. (DOCX)

S2 File English version of the SQS. (DOCX)
