# Supplementary figures and images for: Adaptation and validity of the Sleep Quality Scale among Chinese drivers
Source: PLoS One. 2021 Nov 11;16(11):e0259813. doi: 10.1371/journal.pone.0259813 (PMC8584771; doi:10.1371/journal.pone.0259813)

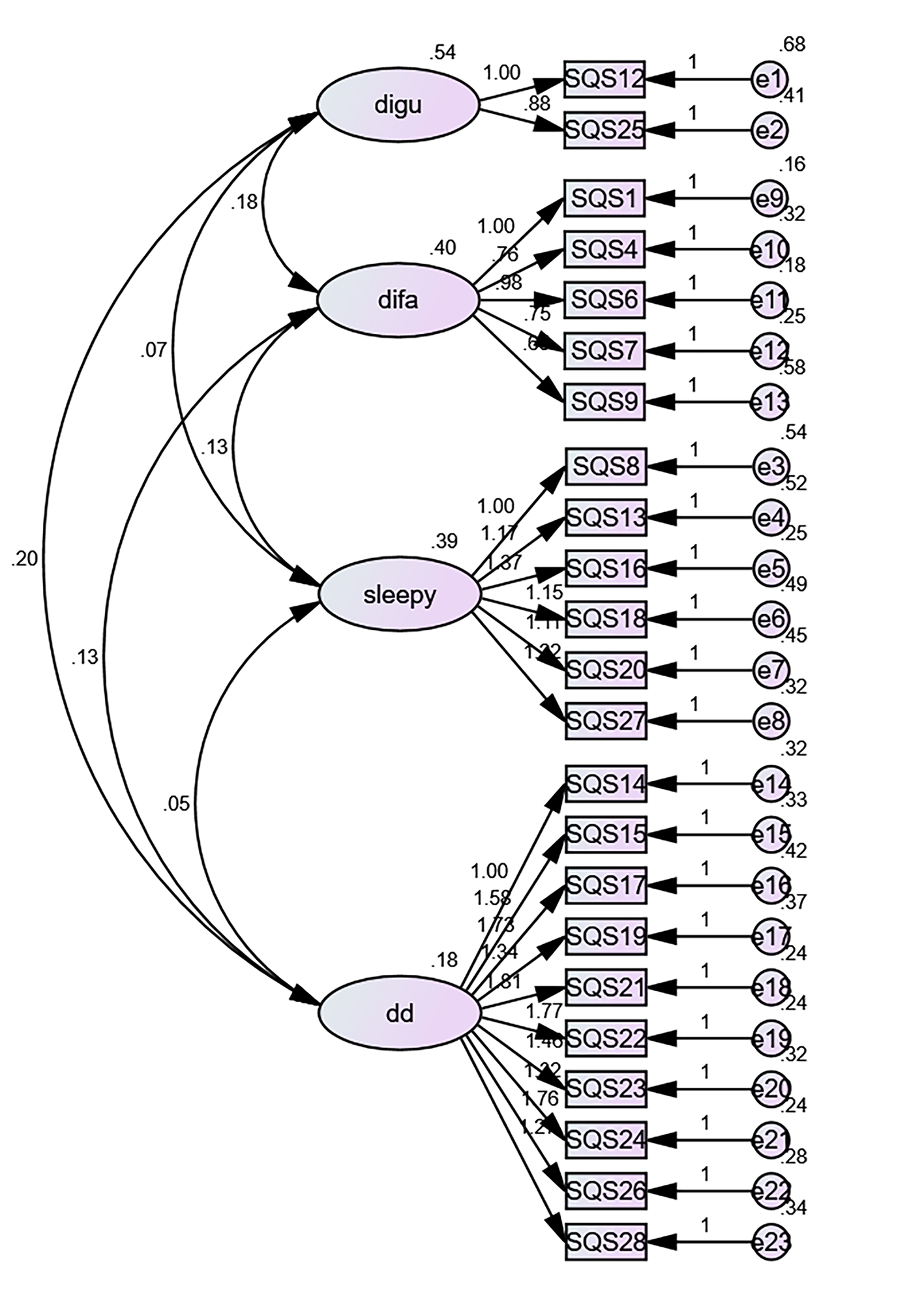

Supplement: S1 Appendix — Note: digu: difficulty in getting up; difa: difficulty in falling asleep; sleepy: sleep recovery; dd: daytime dysfunction. (TIF) [file pone.0259813.s001.tif]
