# Supplementary material for: Adaptation and validity of the Sleep Quality Scale among Chinese drivers
Source: PLoS One. 2021 Nov 11;16(11):e0259813. doi: 10.1371/journal.pone.0259813 (PMC8584771; doi:10.1371/journal.pone.0259813)
Supplement: S1 File — (DOCX) [file pone.0259813.s002.docx]

**睡眠质量量表（SQS）中文版**

指导语：下面一些问题是关于你最近一个月的睡眠质量。所有项目共用答案，请阅读条目并勾选出最适合的答案。选项说明**：**0=很少，1=有时，2=经常，3=几乎总是。

| 1. 我起床后仍需要更多的睡眠 | □0 | □1 | □2 | □3 |
| --- | --- | --- | --- | --- |
| 2. 我睡醒后起床困难 | □0 | □1 | □2 | □3 |
| 3. 我入睡困难 | □0 | □1 | □2 | □3 |
| 4. 我在夜间醒来很难继续入睡 | □0 | □1 | □2 | □3 |
| 5. 我辗转反侧，难以入睡 | □0 | □1 | □2 | □3 |
| 6. 我在醒来不能继续入睡 | □0 | □1 | □2 | □3 |
| 7. 我在睡醒之后不想继续入睡 | □0 | □1 | □2 | □3 |
| 8. 我在睡醒后感到身体轻松 | □0 | □1 | □2 | □3 |
| 9. 我的睡眠时间充足 | □0 | □1 | □2 | □3 |
| 10. 我在睡醒后感到精力充沛 | □0 | □1 | □2 | □3 |
| 11. 我在睡醒后感到疲劳缓解 | □0 | □1 | □2 | □3 |
| 12. 对自己的睡眠感到满意 | □0 | □1 | □2 | □3 |
| 13. 我在睡醒后感到头脑清醒 | □0 | □1 | □2 | □3 |
| 14. 我因睡眠质量差而食欲不振 | □0 | □1 | □2 | □3 |
| 15. 我因睡眠质量差而思考困难 | □0 | □1 | □2 | □3 |
| 16. 我因睡眠质量差而对工作或其他事物失去兴趣 | □0 | □1 | □2 | □3 |
| 17. 我因睡眠质量差而错误增加 | □0 | □1 | □2 | □3 |
| 18. 我因睡眠质量差而容易忘事 | □0 | □1 | □2 | □3 |
| 19. 我因睡眠质量差而难以集中注意力 | □0 | □1 | □2 | □3 |
| 20. 嗜睡影响我的日常生活 | □0 | □1 | □2 | □3 |
| 21. 我因睡眠质量差而失去欲望 | □0 | □1 | □2 | □3 |
| 22. 我因睡眠质量差而在工作中容易感到困倦 | □0 | □1 | □2 | □3 |
| 23. 我因睡眠质量差而感到痛苦 | □0 | □1 | □2 | □3 |
