# Supplementary material for: Adaptation and validity of the Sleep Quality Scale among Chinese drivers
Source: PLoS One. 2021 Nov 11;16(11):e0259813. doi: 10.1371/journal.pone.0259813 (PMC8584771; doi:10.1371/journal.pone.0259813)
Supplement: S2 File — (DOCX) [file pone.0259813.s003.docx]

English version of the SQS

| Items | Few | Sometimes | Often | Almost always |
| --- | --- | --- | --- | --- |
| 1. Refreshed feeling of body after sleep | 0 | 1 | 2 | 3 |
| 2. Enough sleep time | 0 | 1 | 2 | 3 |
| 3. Regaining vigor after sleep | 0 | 1 | 2 | 3 |
| 4. Relief of fatigue after sleep | 0 | 1 | 2 | 3 |
| 5. Satisfaction with sleep | 0 | 1 | 2 | 3 |
| 6. Clear-headed feeling after sleep | 0 | 1 | 2 | 3 |
| 7. Difficulty in falling asleep | 0 | 1 | 2 | 3 |
| 8. Difficulty in getting back to sleep after nocturnal awakening | 0 | 1 | 2 | 3 |
| 9. Tossing and turning sleeplessly | 0 | 1 | 2 | 3 |
| 10. Never falling asleep after awakening during sleep | 0 | 1 | 2 | 3 |
| 11. Feeling unlikely to sleep after sleep | 0 | 1 | 2 | 3 |
| 12. Wish for more sleep after getting up | 0 | 1 | 2 | 3 |
| 13. Difficulty in getting up after sleep | 0 | 1 | 2 | 3 |
| 14. Decrease of appetite due to poor sleep | 0 | 1 | 2 | 3 |
| 15. Difficulty in thinking due to poor sleep | 0 | 1 | 2 | 3 |
| 16. Decrease of interest in work or others due to poor sleep | 0 | 1 | 2 | 3 |
| 17. Increase of mistakes due to poor sleep | 0 | 1 | 2 | 3 |
| 18. Increase of forgetfulness due to poor sleep | 0 | 1 | 2 | 3 |
| 19. Difficulty in concentrating due to poor sleep | 0 | 1 | 2 | 3 |
| 20. Sleepiness that interferes with daily life | 0 | 1 | 2 | 3 |
| 21. Decrease of desire due to poor sleep | 0 | 1 | 2 | 3 |
| 22. Getting tired easily at work due to poor sleep | 0 | 1 | 2 | 3 |
| 23. Painful life due to poor sleep | 0 | 1 | 2 | 3 |
